# Food insecurity and the role of food assistance programs in supporting diet quality during the COVID-19 pandemic in Massachusetts

**DOI:** 10.3389/fnut.2022.1007177

**Published:** 2023-01-05

**Authors:** Matthew M. Lee, Mary Kathryn Poole, Rachel M. Zack, Lauren Fiechtner, Eric B. Rimm, Erica L. Kenney

**Affiliations:** ^1^Department of Nutrition, Harvard T.H. Chan School of Public Health, Boston, MA, United States; ^2^The Greater Boston Food Bank, Boston, MA, United States; ^3^Harvard Medical School, Boston, MA, United States; ^4^Division of General Academic Pediatrics, Department of Pediatrics, MassGeneral Hospital for Children, Boston, MA, United States; ^5^Department of Gastroenterology and Nutrition, MassGeneral Hospital for Children, Boston, MA, United States; ^6^Department of Epidemiology, Harvard T.H. Chan School of Public Health, Boston, MA, United States; ^7^Department of Social and Behavioral Sciences, Harvard T.H. Chan School of Public Health, Boston, MA, United States

**Keywords:** diet quality, food insecurity, COVID-19, nutrition assistance, Massachusetts, food pantry, Supplemental Nutrition Assistance Program (SNAP), lower income

## Abstract

**Background:**

Economic and supply chain shocks resulting from the COVID-19 pandemic in 2020 led to substantial increases in the numbers of individuals experiencing food-related hardship in the US, with programs aimed at addressing food insecurity like the Supplemental Nutrition Assistance Program (SNAP) and food pantries seeing significant upticks in utilization. While these programs have improved food access overall, the extent to which diet quality changed, and whether they helped mitigate diet quality disruptions, is not well understood.

**Objective:**

To evaluate food insecurity, food pantry and/or SNAP participation associations with both diet quality as well as perceived disruptions in diet during the COVID-19 pandemic among Massachusetts adults with lower incomes.

**Methods:**

We analyzed complete-case data from 1,256 individuals with complete data from a cross-sectional online survey of adults (ages 18 years and above) living in Massachusetts who responded to “The MA Statewide Food Access Survey” between October 2020 through January 2021. Study recruitment and survey administration were performed by The Greater Boston Food Bank. We excluded respondents who reported participation in assistance programs but were ineligible (*n* = 168), those who provided straightlined responses to the food frequency questionnaire component of the survey (*n* = 34), those with incomes above 300% of the federal poverty level (*n* = 1,427), those who completed the survey in 2021 (*n* = 8), and those who reported improved food insecurity (*n* = 55). Current dietary intake was assessed *via* food frequency questionnaire. Using Bayesian regression models, we examined associations between pandemic food insecurity, perceived disruption in diet, diet quality, and intakes of individual foods among those who completed a survey in 2020. We assessed interactions by pantry and SNAP participation to determine whether participation moderated these relationships.

**Results:**

Individuals experiencing food insecurity reported greater disruption in diet during the pandemic and reduced consumption of healthy/unhealthy foods. Pantry participation attenuated significant associations between food insecurity and lower consumption of unhealthy (*b* = −1.13 [95% CI −1.97 to −0.31]) and healthy foods (*b* = −1.07 [−1.82 to −0.34]) to null (unhealthy foods: −0.70 [−2.24 to 0.84]; healthy foods: 0.30 [−1.17 to 1.74]), whereas SNAP participation attenuated associations for healthy foods alone (from −1.07 [−1.82 to −0.34] to −0.75 [−1.83 to 0.32]). Results were robust to choice of prior as well as to alternative modeling specifications.

**Conclusion:**

Among adults with lower incomes, those experiencing food insecurity consumed less food, regardless of healthfulness, compared to individuals not experiencing food insecurity. Participation in safety-net programs, including SNAP and pantry participation, buffered this phenomenon. Continued support of SNAP and the food bank network and a focus on access to affordable healthy foods may simultaneously alleviate hunger while improving nutrition security.

## Introduction

Food insecurity (FI), or inconsistent access to enough food to maintain a healthy lifestyle (1), is associated with adverse physical, cognitive, and emotional health (2–4). In the 21st century, FI is often characterized by a state of having reduced access to healthy foods in particular, forcing a reliance on inexpensive but unhealthy or ultra-processed foods and beverages (5, 6).

The economic and supply chain shocks that resulted from the COVID-19 pandemic in 2020 led to a substantial increase in the numbers of individuals experiencing food-related hardship in the US (7). Programs that aim to alleviate FI saw an uptick in participation, including the Supplemental Nutrition Assistance Program (SNAP) and charitable food network system programs, such as pantries (8–11). SNAP provides a monthly cash-like benefit for low-income households to purchase foods once households have completed paperwork to verify eligibility (12), whereas pantries are designed to provide food to anyone at no cost directly, sometimes without requirements to prove income or citizenship status.

In addition to disrupting food supply chains (13), the pandemic resulted in sustained unemployment (14), reducing the purchasing power of many households and increasing reliance on inexpensive, less healthy foods and beverages. While increases in FI and the role of the hunger safety-net during the pandemic are well documented (7, 15), the extent to which COVID-19 has impacted comprehensive diet quality among those at risk for FI is not (16, 17). The pandemic’s impact on diet quality had the potential to be profound with ripple effects that could help explain observed population weight gain from 2020 to 2022 (18, 19). The extent to which diet quality changed, and whether programs designed to alleviate FI like SNAP and pantries helped mitigate diet quality changes, is not well understood, but investigating these questions could help identify how best to support those at risk of nutrition insecurity – defined as lacking consistent access, availability, and affordability of foods and beverages that promote well-being, prevent disease, and treat disease if needed (20, 21).

The purpose of this study was to evaluate the relationships between food insecurity, food pantry participation, SNAP participation, and diet quality (objective quality of foods consumed) and disruption (subjective perceived adverse changes in diet) during the pandemic among Massachusetts adults with low incomes. We hypothesized that diet quality was poor across the population; however, we expected to see lower diet quality and greater disruptions in diet for those reporting FI compared to those who reported experiencing food security, and for those who did not get food from a pantry or did not participate in SNAP. We also hypothesized that participation in pantries or in SNAP would attenuate associations between FI and diet quality and disruption.

## Materials and methods

### Study sample

**FIGURE 1:**
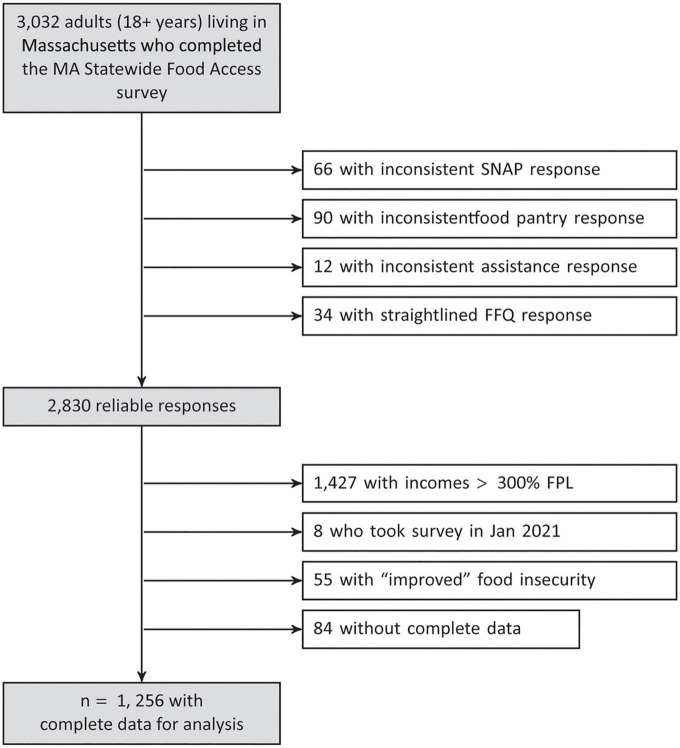
Participant flow diagram. FFQ, food frequency questionnaire; FPL, federal poverty level; GBFB, The Greater Boston Food Bank; MA, Massachusetts; NFACT, National Food Access and COVID Research Team; SNAP, Supplemental Nutrition Assistance Program.

We conducted a cross-sectional analysis among a subset of Massachusetts adults (≥18 years) who participated in an online survey (“The MA Statewide Food Access Survey”) between October 2020 and January 2021 distributed by The Greater Boston Food Bank (GBFB). All respondents who were above the age of 18 years and lived in the state of Massachusetts were eligible to respond to the survey. We recruited participants through multiple market panels using the Qualtrics Panels Project platform. With a target sample size of 3,000 survey respondents, quotas on gender, age, race, ethnicity, educational attainment, and geographic region were set to be proportional to Massachusetts residents 18 years of age and older based on American Community Survey (ACS) 5-year 2019 data. Sample size calculations aimed to provide stable estimates of food access and food insecurity overall and by key sociodemographics. In particular, participants with lower incomes were oversampled to provide greater statistical power for comparisons among this group, including users and non-users of pantries (22). Additional details on study design are also published elsewhere (22, 23). We excluded respondents whose surveys did not pass a data quality check for inconsistent or illogical answers (Figure 1, *n* = 202). These included individuals who reported participation in federal assistance programs but were ineligible (*n* = 168), and those who provided straightlined responses to the food frequency questionnaire (*n* = 34). Because relationships between program participation and diet could be confounded by household income, which is a strong predictor of diet quality, we also excluded those with household incomes above 300% of the federal poverty level (FPL) (*n* = 1,427) to minimize the potential for residual confounding after income adjustment. We also excluded those who completed the survey in 2021 (*n* = 8) or those who reported improved FI (*n* = 55) due to small sample sizes, as well as 84 individuals with missing data. We also excluded individuals who reported improved food security as our primary research question was whether participation in SNAP or in food pantries moderated associations between experiencing food insecurity and diet outcomes for those who experienced food insecurity during the pandemic compared to those who did not at any point. The final analytic sample comprised 1,256 individuals. The study was approved by the Harvard T.H. Chan School of Public Health and D’Youville College Institutional Review Boards (date of approval: April 4, 2021; ID: DAT21-0286).

### Measures

Respondents completed a survey featuring a series of closed-ended questions about access to and types of foods consumed pre-pandemic (between March 11, 2019 and March 11, 2020) and during the pandemic (between March 11, 2020 and the survey date). The survey was modified from a survey created by the National Food Access and COVID Research Team (NFACT) (24), which demonstrated good internal consistency and reliability in a previous pilot study in Vermont (25, 26).

#### Primary outcome: Diet quality

Diet quality was measured in the survey using a modified version of the Prime Diet Quality Score (PDQS), a validated tool that has been used as a brief, low-burden approach to capture overall diet quality (27, 28). Content validity for this measure and its scoring was assessed *via* expert consultation with the original creators of the PDQS. Respondents indicated how frequently they consumed each of 14 foods in the last 30 days: (1) processed meats; (2) beef, pork, lamb; (3) fish; (4) full fat dairy products; (5) fast food or take-out, pizza, frozen dinners, restaurant meals; (6) soda, soft drinks, sports or energy drinks; (7) white bread, white rice, white pasta; (8) whole grain bread, brown rice, whole grain pasta; (9) sweets and desserts; (10) beans, lentils, chickpeas, tofu; (11) vegetables; (12) whole fruits; (13) peanut butter and nuts; and (14) beer, wine, or liquor. Response options included less than once per week, once/week, 2–4 times/week, nearly daily or daily, or twice or more per day. We generated three aggregate measures to obtain measures of overall diet quality. First, we computed an “Overall Diet Quality” measure by assigning a point to each food frequency response, consistent with prior work (28). For healthy items (fruits, vegetables, whole grains, nuts, legumes, and fish), we assigned a value between 1 and 5 with higher points indicating *more* frequent consumption. For unhealthy items (processed meat, beef/pork/lamb, fast food, SSBs, sweets, refined grains, full-fat dairy, and alcohol), we assigned a value between 1 and 5 with higher points indicating *less* frequent consumption. The final Overall PDQS (out of 70) measure was computed as the sum across all items, with higher scores indicating better diet quality. Second, we computed “Unhealthy PDQS” (out of 40) and “Healthy PDQS” (out of 30) sub-measures by assigning a point value between 1 and 5 with higher points indicating *more* frequent consumption, then summing scores within each category of unhealthy or healthy foods. Higher Unhealthy PDQS values indicate poorer diet quality whereas higher Healthy PDQS values indicate better diet quality. For food-specific models, we examined consumption frequency on a continuous scale by converting all responses to times/week, using the midpoint of each interval and top-coding “twice or more per day” as 10 times/week and bottom-coding “less than once per week” response as 0.5 times/week.

#### Secondary outcome: Dietary disruption

Respondents also were asked to self-report, for each of the food/beverage categories, whether they were eating less, did not change, or were eating more compared to before the pandemic. We summed these responses to create an overall measure of perceived disruption in diet, adding a point if a respondent answered “eating less” for each healthy food or “eating more” for each unhealthy food, and adding zero points otherwise. Thus, higher diet disruption scores indicated participants perceived their diets to have changed for the worse. Overall scores on the disruption score ranged from 0 to 14, with high scores indicating greater disruption in overall diet.

#### Exposures: Food security and pantry/SNAP participation status

Participants completed the USDA 6-item Short-Form Food Security Module (29), both retrospectively for the year prior to the pandemic and for the 30 days prior to the survey. Respondents also specified whether they had used a pantry and whether they have participated in SNAP in the past 30 days.

#### Covariates

Respondents reported basic sociodemographic information including zip code, age (in years), gender (male/female), race/ethnicity (non-Hispanic White, Hispanic/Latino or Spanish origin, non-Hispanic Black or African American, non-Hispanic Asian, Other), highest educational attainment (High school or less, High school graduate, Some college, Associate’s degree, Bachelor’s degree, Graduate degree), household income (<$10K, $10–$25K, $150–$200K, $25K–$50K, $50K–$75K, $75K–100K, ≥$200K), household size, whether there were children in the household, current employment status, and experience of household job disruption since the pandemic. Information on household income and size was used to determine highest possible household income as a percent of the federal poverty level (% FPL) based on published 2021 US thresholds for each respondent, which we then converted into a categorical variable (≤100% FPL, 100–200% FPL, 200–300% FPL). Respondents also reported Special Supplemental Nutrition Program for Women, Infants, and Children (WIC) and school meals program participation in the past month, self-rated poor/fair health, and poor mental wellbeing. Self-rated health was measured using the prompt, “In general, would you say your health is:” with response options given by “Excellent,” “Very good,” “Good,” “Fair,” or “Poor.” Mental wellbeing was assessed *via* the two-item version of the Patient Health Questionnaire (PHQ). Respondents rated how often (not at all, several days, more than half the days, or nearly every day), over the last 2 weeks, they had been bothered with “little interest or pleasure in doing things” or “feeling down, depressed, or hopeless.” Scores for each item ranged from 0 to 3, with 0 points assigned for “Not at all” and 3 points assigned for “Nearly every day.” We summed scores for the two items and created an indicator for poor mental wellbeing defined as values of the summed score greater than or equal to 3. Neighborhood-level household median income, proportion below 185% FPL, and proportion without a high school education/diploma were obtained by linking participants’ zip codes with Census Zip Code Tabulation Area estimates from the 2019 5-year American Community Survey.

### Statistical analysis

We assessed crude differences in key variables, including diet quality, dietary intake, nutrition assistance program participation, survey response, race/ethnicity, age, gender, educational attainment, employment status and job disruption, self-rated health, and mental wellbeing, between those who always experienced food security to those who experienced food insecurity at any point. We tested for differences using continuity-corrected chi-squared tests for categorical variables, and using two-group analysis of variance for continuous variables (Table 1). In models for mean differences in diet quality and in perceived diet disruption (Overall PDQS/Disruption, Unhealthy PDQS, and Healthy PDQS), we estimated parameters from Bayesian linear regression models that first included terms for FI, SNAP participation, pantry participation, and covariates, to test for whether FI and food assistance receipt were independently associated with diet quality or disruption. Covariates in these models included: gender, age, race/ethnicity, highest educational attainment, household size, presence of children in the household, current employment status, experience of job disruption, income as a% of the federal poverty level, month the survey was taken, WIC or school meals participation in the past month, self-rated poor or fair health, poor mental wellbeing, and neighborhood median income, proportion below 185% FPL, and proportion without a high school diploma. We then estimated a model that additionally tested terms for the interaction between FI and SNAP participation and the interaction between FI and pantry participation to test whether associations between FI and diet quality were modified by SNAP or pantry use. Bayesian estimation allows researchers to encode information from both the observed data as well as from prior knowledge *via* a distributional assumption related to the likely values of the parameters (30). For all analyses, we used informative null prior distributions, thereby addressing multiple comparisons concerns by regularizing parameter estimates toward the null (31). For PDQS and Disruption models, we used Normally distributed priors with a mean of 0 and standard deviation of 1. In exploratory analyses, we estimated parameters from log-linear models to obtain adjusted consumption frequency ratios for each of the 14 food items, using the predictors as that of the PDQS models. These regularizing priors provided control against multiple testing in the estimation process, as compared to frequentist methods that control for multiple testing during *post hoc* adjustment of *p*-values (32), by shrinking estimates toward the null. Non-null results are therefore more likely to represent strong signals from the data of an association above and beyond random chance.

**TABLE 1:** Sociodemographic and dietary characteristics of Massachusetts adults with household incomes < 300% of the federal poverty line in the MA statewide food access survey, Oct 2020 – Dec 2020 (*n* = 1,256).

	Persistently food secure (*n* = 489)	Food insecure at any point (*n* = 767)	*P*-value^1^
**Prime diet quality screener (PDQS) scores [mean ± SD]**			
Overall PDQS score (out of 70, higher is better)	44.63 ± 5.92	43.60 ± 5.16	0.002
Healthy PDQS score (out of 30, higher is better)	14.71 ± 3.99	14.02 ± 4.16	0.003
Unhealthy PDQS score (out of 40, higher is worse)	18.08 ± 4.91	18.42 ± 4.94	0.23
**Individual food item frequencies (times/week) [mean ± SD]^2^**			
Fruits	4.0 ± 2.9	3.1 ± 2.7	<0.001
Vegetables	4.4 ± 2.8	3.4 ± 2.8	<0.001
Whole grains	2.5 ± 2.4	2.4 ± 2.3	0.71
Nuts	2.8 ± 2.6	2.7 ± 2.6	0.70
Legumes	1.8 ± 2.1	2.0 ± 2.3	0.14
Fish	1.3 ± 1.5	1.4 ± 1.8	0.27
Processed meat	1.8 ± 2.0	2.3 ± 2.3	<0.001
Beef, pork, lamb	2.2 ± 2.1	2.1 ± 2.2	0.28
Fast food, takeout	1.6 ± 1.9	2.0 ± 2.2	0.009
Sugar-sweetened beverages (SSBs)	2.6 ± 3.0	2.8 ± 2.9	0.24
Sweets	2.9 ± 2.6	2.4 ± 2.5	0.005
Refined grains	3.1 ± 2.7	3.4 ± 2.6	0.05
Full-fat dairy	3.8 ± 2.8	3.5 ± 2.8	0.07
Alcohol	1.4 ± 2.0	1.5 ± 2.1	0.95
**Participation in nutrition assistance programs**			
Participated in SNAP in past month [*N* (%)]	107 (21.9)	280 (36.5)	<0.001
Participated in WIC in past month [*N* (%)]	18 (3.7)	59 (7.7)	0.006
Participated in food pantries/Banks in past month [*N* (%)]	32 (6.5)	155 (20.2)	<0.001
Participated in school meals during pandemic [*N* (%)]	57 (11.7)	217 (28.3)	<0.001
Month survey taken [*N* (%)]			0.007
October 2020	104 (21.3)	110 (14.3)	
November 2020	218 (44.6)	370 (48.2)	
December 2020	167 (34.2)	287 (37.4)	
**Individual demographic characteristics**			
Race/Ethnicity [*N* (%)]			0.004
Non-hispanic white	351 (71.8)	504 (65.7)	
Non-hispanic black	41 (8.4)	73 (9.5)	
Hispanic	48 (9.8)	129 (16.8)	
Non-hispanic Asian	31 (6.3)	31 (4.0)	
Other race/Ethnicity	18 (3.7)	30 (3.9)	
Age (years) [mean ± SD]	43.2 ± 18.1	35.1 ± 13.6	<0.001
Gender [*N* (%)]			0.13
Male	148 (30.3)	201 (26.2)	
Female	341 (69.7)	566 (73.8)	
Educational attainment [*N* (%)]			<0.001
High school or less	19 (3.9)	53 (6.9)	
High school graduate (including GED)	150 (30.7)	255 (33.2)	
Some college (no degree)	106 (21.7)	220 (28.7)	
Associates degree	60 (12.3)	102 (13.3)	
Bachelor’s degree	112 (22.9)	103 (13.4)	
Graduate degree	42 (8.6)	34 (4.4)	
Currently employed [*N* (%)]	251 (51.3)	397 (51.8)	0.93
Experienced job disruption during pandemic [*N* (%)]	203 (41.5)	500 (65.2)	<0.001
Self-rated fair or poor health [*N* (%)]	111 (22.7)	262 (34.2)	<0.001
Patient health questionnaire (PHQ-6) score [mean ± SD]	1.7 ± 1.9	3.2 ± 2.0	<0.001
**Household characteristics**			
Poverty [*N* (%)]			<0.001
Less than or equal to 100% FPL	85 (17.4)	248 (32.3)	
100–200% FPL	170 (34.8)	298 (38.9)	
200–300% FPL	234 (47.9)	221 (28.8)	
Household size (people) [mean ± SD]	3.2 ± 1.9	3.7 ± 2.3	<0.001
Children are present in the household [*N* (%)]	159 (32.5)	387 (50.5)	<0.001
**Neighborhood (census zip code tabulation area) characteristics [mean ± SD]**			
Median household income ($, thousands)	17.98 ± 10.66	18.98 ± 10.78	0.11
Proportion below 185% federal poverty level	0.24 ± 0.12	0.27 ± 0.13	<0.001
Proportion without high school education/Diploma	0.37 ± 0.14	0.41 ± 0.14	<0.001

^1^*P*-values are from continuity-corrected chi-squared tests for categorical variables, and from two-group analysis of variance for continuous variables.

^2^Food frequency values reflect consumption of the total population.

FPL, federal poverty level; GBFB, Greater Boston Food Bank; GED, general education development; MA, Massachusetts; NFACT, National Food Access and COVID Research Team; PDQS, Prime Diet Quality Score; SNAP, Supplemental Nutrition Assistance Program; WIC, Special Supplemental Nutrition Program for Women, Infants, and Children.

We used survey weights in all analyses. Point estimates were taken to be the mean, and 95% credible intervals were taken to be the 2.5th and 97.5th percentile of the posterior distribution for each parameter. We calculated Bayesian p-values using the probability of direction (pd) from the posterior samples (33). We assessed statistical significance using a nominal type I error rate of 0.05. We used Hamiltonian Markov Chain Monte Carlo (MCMC) with 10,000 iterations across four chains and confirmed adequate mixing and convergence. All analyses were conducted in R 4.0.5 *via* the *brms* package.

### Sensitivity analyses

We tested the sensitivity of our results to choice of prior (e.g., increasing the standard deviation of the prior 5 and 10 fold) and against random effects models with random county- or region-level intercepts. Variability of the random-intercepts was very small, so we excluded random effects from the final model. We also re-fit all models with non-informative Uniform priors, which produces equivalent estimates to that of standard maximum likelihood estimation (30). While point estimates from these models were slightly larger in magnitude (as expected with a non-informative prior), statistical significance and 95% credible intervals produced the same set of conclusions. Results were also similar when excluding respondent alcohol consumption, which we tested based on evidence suggesting potential benefits of moderate intake on cardiometabolic health (34).

## Results

### Sample demographics

In this sample of adults with lower incomes in Massachusetts, a majority of respondents identified as non-Hispanic White race/ethnicity (Food Secure: 71.8%, Food Insecure: 65.7%), with higher proportions in the group experiencing food insecurity identifying as non-Hispanic Black (Food Secure: 8.4%, Food Insecure: 9.5%), Hispanic (Food Secure: 9.8%, Food Insecure: 16.8%), or some other race/ethnicity (Food Secure: 3.7%, Food Insecure: 3.9%) (Table 1). More than half (61.0%) identified as food insecure. Those who were experiencing FI were significantly (*p* < 0.05) more likely to have lower educational attainment (Food Secure: 56.3%, Food Insecure: 68.8% without a college degree), have experienced job disruption during the pandemic (Food Secure: 41.5%, Food Insecure: 65.2%), report fair/poor self-rated health (Food Secure: 22.7%, Food Insecure: 34.2%), and report poor mental wellbeing (Food Secure: 1.7, Food Insecure: 3.2 mean PHQ-2 score). In addition, those who were experiencing FI were more likely to have incomes < 100% FPL (32.4%) compared to their food secure counterparts (17.4%) and were more likely to have children living in the household (Food Secure: 32.5%, Food Insecure: 50.5%). Respondents experiencing FI were also more likely to report participating in SNAP in the past month (Food Secure: 21.9%, Food Insecure: 36.5%), WIC (Food Secure: 3.7%, Food Insecure: 7.7%), food pantries (Food Secure: 6.5%, Food Insecure: 20.2%), and school meals (Food Secure: 11.7%, Food Insecure: 28.3%).

### Perceived disruption in diet

Regardless of pantry or SNAP participation status, individuals experiencing FI reported greater perceived disruption in overall diet due to the COVID-19 pandemic compared to their food secure counterparts (Table 2), after adjustment for covariates (Among non-participants of both programs: 1.25 [0.80 to 1.70]; Among food pantry participants alone: 1.63 [95% CrI: 0.57 to 2.69]; Among SNAP participants alone: 0.94 [0.27 to 1.60]; Among participants of both food pantries and SNAP: 1.32 [0.27 to 2.38]). This was primarily driven by perceived reduced consumption of healthy foods.

**TABLE 2:** Adjusted^1^ mean diet disruption and PDQS score differences, by SNAP, food pantry, and food insecurity status, among low-income Massachusetts adults (*n* = 1,256), Oct 2020 – Jan 2021.

	Overall diet quality (PDQS)^2^		Healthy diet score (PDQS)^3^		Unhealthy diet score (PDQS)^4^		Perceived dietary disruption score^5^	
	b (95% CrI)	*P*-value^6^	b (95% CrI)	*P*-value^6^	b (95% CrI)	*P*-value^6^	b (95% CrI)	*P*-value^6^
Food insecure	0.33 (−0.51, 1.16)	0.44	−0.86 (−1.58, −0.16)	0.02	−1.17 (−1.99, −0.40)	0.003	1.19 (0.78, 1.58)	<0.001
SNAP participation in last month	−0.48 (−1.29, 0.33)	0.25	0.48 (−0.20, 1.14)	0.16	0.98 (0.22, 1.74)	0.01	−0.46 (−0.85, −0.07)	0.02
Food pantry participation in last month	0.60 (−0.43, 1.64)	0.25	1.23 (0.33, 2.10)	0.01	0.53 (−0.41, 1.48)	0.29	0.41 (−0.11, 0.92)	0.12
**Interactions between food insecurity and program participation**								
Food insecure compared to food secure, no program participation	0.14 (−0.74, 1.03)	0.77	−1.07 (−1.82, −0.34)	<0.001	−1.13 (−1.97, −0.31)	<0.001	1.25 (0.80, 1.70)	<0.001
Food insecure compared to food secure, food pantry participation only	0.87 (−0.74, 2.50)	0.29	0.30 (−1.17, 1.74)	0.68	−0.70 (−2.24, 0.84)	0.36	1.63 (0.57, 2.69)	<0.001
Food insecure compared to food secure, SNAP participation only	0.64 (−0.61, 1.89)	0.32	−0.75 (−1.83, 0.32)	0.16	−1.40 (−2.58, −0.20)	0.02	0.94 (0.27, 1.60)	<0.001
Food insecure compared to food secure, SNAP and pantry participation combined	1.37 (−0.36, 3.08)	0.12	0.62 (−0.94, 2.16)	0.44	−0.97 (−2.59, 0.69)	0.24	1.32 (0.27, 2.38)	0.01

CrI, credible interval; PDQS, Prime Diet Quality Score; SNAP, Supplemental Nutrition Assistance Program.

^1^Models included terms for: food insecurity status; participation in SNAP, participation in food pantries; and the following covariates: gender, age, race/ethnicity, highest educational attainment, household size, presence of children in the household, current employment status, experience of job disruption, income as a% of the federal poverty level, month the survey was taken, WIC or school meals participation in the past month, self-rated poor or fair health, poor mental wellbeing, and neighborhood median income, proportion below 185% FPL, and proportion without a high school diploma.

^2^Overall PDQS is out of 70, higher scores indicate better diet quality.

^3^Healthy PDQS is out of 40, higher scores indicate worse diet quality.

^4^Unhealthy PDQS is out of 30, higher scores indicate better diet quality.

^5^Overall disruption score is out of 14, with higher scores indicating greater disruption in overall diet.

^6^*P*-values are computed from probability of direction (pd) values based on posterior samples.

### Diet quality

**FIGURE 2:**
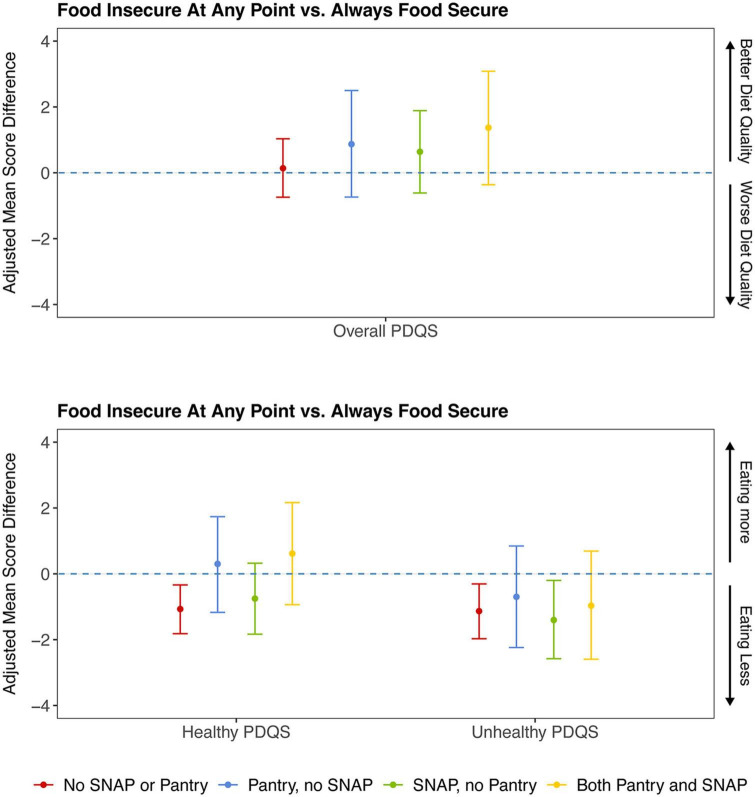
Prime Diet Quality Score^1^, adjusted mean differences^2^, by program participation and food insecurity status. ^1^Three types of Prime Diet Quality Scores were assessed as outcomes: overall, healthy, and unhealthy. For the overall scale (out of 70), higher scores indicate higher frequencies of healthy items and lower frequencies of unhealthy items. For the Healthy scale (out of 30), higher scores indicate higher frequencies of healthy items. For the Unhealthy scale (out of 40), higher scores indicate higher frequencies of unhealthy items. A point estimate that does not include the null value (0) indicates a significant adjusted mean difference in diet quality score comparing those who are food insecure to those who were always experiencing food security for a specific stratum of program participation. For example, the first red line under the “Healthy PDQS” collection of estimates indicates that individuals who were food insecure at any point consumed less healthy items than those who were experiencing food security at all time points on average, among those who did not participate in SNAP or in food pantries within the last month, adjusted for covariates. ^2^Mean differences shown are adjusted for gender, age, race/ethnicity, highest educational attainment, household size, presence of children in the household, current employment status, experience of job disruption, income as a% of the federal poverty level, month the survey was taken, WIC or school meals participation in the past month, self-rated poor or fair health, poor mental wellbeing, and neighborhood median income, proportion below 185% FPL, and proportion without a high school diploma. Models were weighted by MA Statewide Food Access survey weights. PDQS, Prime Diet Quality Score; SNAP, Supplemental Nutrition Assistance Program.

Adjusting for sociodemographic factors, when examining the association between FI and overall diet quality (overall PDQS score), there appeared to be no relationship (Figure 2 and Table 2). However, this overall null relationship obscured differences observed when examining Healthy and Unhealthy sub-scores—participants experiencing FI had significantly lower scores for both types of sub-scores, suggesting they ate less of both healthy and unhealthy items. Meanwhile, using a pantry, regardless of food security status, was associated with better diet quality indicated by a significantly higher Healthy PDQS sub-score (+1.23, 95% CrI 0.33 to 2.10), while using SNAP was associated with worse diet quality indicated by a significantly higher Unhealthy PDQS sub-score (+0.98, 95% CrI 0.22 to 1.74).

When evaluating interactions between food security and pantry and/or SNAP participation to assess whether program participation might change the associations between food security and diet quality, there continued to be no significant differences between those experiencing FI and those that were experiencing food security for the Overall PDQS score, regardless of program. Differences in the association between food security and diet quality by program participation were seen, however, in the Healthy PDQS and Unhealthy PDQS sub-scores. Among those who did not participate in either program (neither SNAP nor pantries), individuals experiencing FI had lower unhealthy PDQS scores compared to their food secure counterparts (difference of 1.13 (95% CrI: 0.31 to 1.97), indicating *lowered* consumption of unhealthy items for those who were experiencing FI. Among those who participated in SNAP but did not use pantries, scores for the Unhealthy PDQS were 1.40 (95% CrI: 0.20 to 2.58) points lower on average among those who were experiencing FI compared to those who were experiencing food security, indicating lowered consumption of unhealthy items for those experiencing FI but who participated in SNAP alone. Among those who participated in pantries (with or without SNAP), there were no associations between FI and mean Unhealthy PDQS score, i.e., food insecure respondents who used pantries had similar levels of unhealthy food consumption as those who were experiencing food security.

A similar pattern emerged for the Healthy PDQS score. Individuals experiencing FI who did not participate in SNAP or in pantries had Healthy PDQS scores that were 1.07 (95% CrI: 0.34 to 1.82) points lower (indicating *lowered* consumption of healthy items) compared to those who were experiencing food security. However, any program participation (i.e., in SNAP or pantries together or in isolation), attenuated the association between FI and lowered consumption of healthy foods, such that the Healthy PDQS scores for participants experiencing FI were not different from those who were experiencing food security if they used SNAP or pantries.

### Intake of specific food categories

Compared to those who were always food secure, those that experienced FI at any point consumed significantly less fruits, vegetables, and sweets, and significantly more processed meats, fast food/takeout, and refined grains (Table 1). Individuals experiencing FI who did not participate in SNAP or pantries consumed less fruits (−26.1% [95% CrI: −10.3% to −39.4%]), vegetables (−30.7% [−15.8% to −42.9%]), whole grains (−21.9% [−3.40% to −36.5%]), and nuts (−20.8% [−2.1% to −35.7%]), but also less red meats (−17.2% [−0.20% to −31.1%]), sweets (−26.2% [−9.6% to −39.7%]), and full-fat dairy (−23.1% [−6.0% to −37.7%]) than those experiencing food security. However, those experiencing FI that used pantries had similar levels of consumption of these foods compared to respondents experiencing food security (Supplementary Appendices 1, 2). Meanwhile, individuals experiencing food insecurity who participated in SNAP had similar levels of consumption as individuals experiencing food security for whole grains and nuts, but not other foods.

## Discussion

In this representative study of adults with lower incomes living in Massachusetts during the COVID-19 pandemic in 2020, we found that those who experienced FI at any point in the year prior to or in the past 30 days of the pandemic were more likely to report significant disruptions in their diet compared to those who were experiencing food security, and that participants perceived that they were consuming smaller amounts of healthy items compared to before the pandemic. We also found significant differences in diet quality by food security status. These differences were only observable, however, when examining Healthy and Unhealthy PDQS sub-scores and when examining changes in specific foods. Using pantries buffered the associations between FI and poor diet quality by attenuating associations between FI and lower Healthy and Unhealthy PDQS scores among non-participants of both programs. In contrast, SNAP participation without pantry participation attenuated the association between FI and lower Healthy PDQS score only.

Overall, these findings indicate that individuals experiencing food insecurity consumed fewer foods across both healthy and unhealthy items during the past month in late 2020, and that participation in pantries buffered this phenomenon across the various food groups included in the PDQS. Similar findings were recently reported in a study of fruit and vegetable consumption in the state of Vermont where individuals experiencing food insecurity reported greater disruptions in fruit and vegetable intake, and pantry participation buffered the association between FI and low intake (26). In this Massachusetts-based population, we found that pantry participation attenuated associations between FI and lowered consumption of fruit and vegetables, as well as for red meat, sweets, and dairy. Our results were consistent with the portfolio of foods shipped by the GBFB to pantries in 2020 (Supplementary Appendix 3), which included fruits and vegetables as well as dairy and cereal grain products. Food banks and pantries may be uniquely situated to provide foods to individuals and families experiencing FI during periods of unanticipated loss of income or crisis (9, 35, 36). When these institutions can prioritize the distribution of healthful foods, they then play an important role in minimizing negative impacts of FI on diet quality. While GBFB prioritizes nutrition education and diet as part of their broader goals (37), nutrition is not always a key goal throughout the charitable food system, with prior studies suggesting a high degree of variability in the nutritional quality of pantry offerings (38–40). Strengthening strategies already used by many food banks and pantries, including the GBFB, such as adding a stoplight system to signify the nutritional content of foods on pantry shelves for pantry users (41), adding detailed nutritional information to ordering forms provided by food banks to pantries (42), or providing nutrition counseling training for pantry staff (43) may improve the nutritional quality of participants’ food bags while maintaining autonomy and choice.

SNAP participation also appeared to alleviate some of the associations between FI and poorer diet quality, although not as consistently as pantry participation. In this population, SNAP participation attenuated associations between FI and lowered consumption of whole grains, nuts, sweets, and full-fat dairy. Prior research has found that, compared to eligible non-participants, those enrolled in SNAP experience lower diet quality and may need to purchase foods that are inexpensive but are calorically dense and nutrient-poor in order to maximize their benefits (44). Notably, however, we found in this sample that SNAP participation was *not* associated with increased intake of SSBs, and other recent work suggests that food insecurity is not associated with increased intake of ultra-processed foods among SNAP participants (6). Policymakers should continue to consider strategies aimed to incentivize purchases of healthier foods using SNAP benefits, such as providing financial incentives for fruits and vegetables (45) or increasing the SNAP benefit size (46, 47).

Food insecurity and poor diet quality are not only a concern for chronic diseases and mental health; they may also worsen the health impacts of COVID-19. Recent findings suggests that low diet quality and living in neighborhoods with high socioeconomic deprivation may work to exacerbate risk and severity of COVID-19 infection (48). To promote population health, programs to reduce hunger should consider strategies for increasing the affordability of healthy foods through SNAP and the charitable food system in order to maximize the health of an already vulnerable population.

### Limitations

Despite the strengths of this study, which included a representative sample of Massachusetts residents, and use of validated measures of diet and FI status, several limitations should be noted. First, as with any observational study of cross-sectional data, the presence of unmeasured confounding limits our ability to make causal statements linking pantry or SNAP participation to the effect of FI on diet. Our assessment of diet disruption also relied on recalls of intake prior to the pandemic and is subject to recall bias. We did not assess information on portion size as part of the modified PQDS and were unable to adjust for total energy intake in the analysis. Therefore, our results reflect differences in the absolute quantity of foods consumed, which is of critical importance during public health emergencies, but we could not assess impacts on diet quality that were independent of quantity. Third, we excluded individuals who experienced improved food insecurity status during the pandemic. Future research should assess drivers of improved food insecurity in these populations with the adequate sample size needed for precise statistical testing. Last, we were unable to estimate associations by race/ethnicity or by degree of FI.

## Conclusions and implications

Food insecurity remains a pressing problem for millions of Americans with low-incomes 2 years into the pandemic as the anticipated financial impacts of the pandemic are likely to be ongoing. Safety nets including SNAP and the charitable food network can alleviate hunger while providing opportunities to improve diet quality. We found that participation in pantries or SNAP decreased the likelihood of consuming less food for individuals experiencing FI compared to their food secure counterparts, though this occurred for both healthy and unhealthy items. To improve nutrition security, anti-hunger and public health advocates must find ways to ensure that vulnerable populations have access to healthy, adequate food. During public health emergencies, this may include bolstering existing benefits through programs such as the Pandemic Electronic Benefit Transfer (49), the Child Tax Credit (50), or relaxing eligibility criteria for other key nutrition assistance programs such as free or reduced-priced school meals (51). Continued monitoring and surveillance of population FI and diet quality throughout COVID-19 may help policymakers identify key groups at risk for developing severe disease.

## Data availability statement

The datasets presented in this article are not readily available because data requests must be made to The Greater Boston Food Bank Research Division. Requests to access the datasets should be directed to The Greater Boston Food Bank (https://www.gbfb.org/contact-us/).

## Ethics statement

The studies involving human participants were reviewed and approved by the Harvard T.H. Chan School of Public Health Institutional Review Board and the D’Youville College Institutional Review Board. The patients/participants provided their written informed consent to participate in this study.

## Author contributions

ML, MP, RZ, LF, ER, and EK designed the research. ML analyzed the data and performed the statistical analysis. ML, MP, and EK wrote the manuscript. MP, RZ, LF, ER, and EK provided the critical interpretation and review of the manuscript. All authors have read and approved the final manuscript.
