# Supplementary material for: Food insecurity and the role of food assistance programs in supporting diet quality during the COVID-19 pandemic in Massachusetts
Source: Front Nutr. 2023 Jan 5;9:1007177. doi: 10.3389/fnut.2022.1007177 (PMC9849926; doi:10.3389/fnut.2022.1007177)
Supplement: Supplementary file 1 [file Data_Sheet_1.docx]

**SUPPLEMENT FOR:** Food insecurity and the role of food assistance programs in supporting diet quality during the COVID-19 pandemic in Massachusetts

**CONTENTS**

**APPENDIX 1:** Adjusted consumption frequency ratios for individual foods comparing food insecure at any point vs. Persistently food secure, by program participation (Table)

**APPENDIX 2:** Adjusted consumption frequency ratios for individual foods comparing food insecure at any point vs. Persistently food secure, by program participation (Figure)

**APPENDIX 3:** Foods shipped to local food pantries by the Greater Boston Food Bank from January 1, 2020, through December 31, 2020 (Table)

**APPENDIX 1A:** Adjusted consumption frequency ratios for individual foods comparing program participants to non-participants

|  | **Food Pantries:**  Participants vs. Non-participants, by Food Security | | **SNAP:**  Participants vs. Non-participants, by Food Security | |
| --- | --- | --- | --- | --- |
| Food item | Not Food Insecure  (95% CrI) | Food Insecure  (95% CrI) | Not Food Insecure  (95% CrI) | Food Insecure  (95% CrI) |
| **Healthy items** |  |  |  |  |
| *Fruit* | 0.775 (0.487, 1.232) | 1.189 (0.929, 1.522) | 1.137 (0.874, 1.481) | 1.12 (0.921, 1.362) |
| *Vegetables* | 0.795 (0.503, 1.261) | **1.421 (1.106, 1.826)** | 1.118 (0.858, 1.45) | 1.059 (0.871, 1.291) |
| *Whole Grains* | 0.785 (0.473, 1.291) | 1.138 (0.868, 1.482) | 1.009 (0.761, 1.339) | 1.228 (0.985, 1.525) |
| *Nuts* | 0.822 (0.512, 1.317) | **1.435 (1.113, 1.859)** | 1.05 (0.788, 1.392) | 1.216 (0.984, 1.508) |
| *Legumes* | 0.851 (0.54, 1.352) | **1.526 (1.19, 1.948)** | 0.898 (0.688, 1.171) | 0.996 (0.815, 1.213) |
| *Fish* | **1.601 (1.09, 2.378)** | **1.362 (1.11, 1.662)** | 1.082 (0.865, 1.357) | 0.989 (0.836, 1.169) |
| **Unhealthy items** |  |  |  |  |
| *Processed Meats* | 1.254 (0.798, 1.943) | 1.207 (0.957, 1.53) | 1.094 (0.859, 1.402) | 0.995 (0.822, 1.204) |
| *Beef, Pork, Lamb* | 0.866 (0.553, 1.341) | 1.039 (0.821, 1.315) | **1.408 (1.092, 1.821)** | 1.078 (0.892, 1.312) |
| *Fast Food* | 1.09 (0.698, 1.694) | 0.958 (0.764, 1.211) | 1.095 (0.848, 1.406) | 1.074 (0.892, 1.3) |
| *SSBs* | 0.764 (0.445, 1.298) | 1.071 (0.799, 1.425) | **1.388 (1.016, 1.901)** | 1.073 (0.849, 1.35) |
| *Sweets* | 0.779 (0.473, 1.273) | 0.986 (0.759, 1.275) | 1.086 (0.816, 1.454) | 1.149 (0.93, 1.415) |
| *Refined Grains* | 0.851 (0.53, 1.361) | 1.086 (0.845, 1.402) | **1.34 (1.021, 1.766)** | 1.122 (0.906, 1.384) |
| *Full-Fat Dairy* | 1.101 (0.685, 1.757) | 1.075 (0.826, 1.389) | 1.206 (0.918, 1.596) | 1.231 (0.992, 1.522) |
| *Alcohol* | 1.116 (0.736, 1.699) | **1.252 (1.001, 1.567)** | 0.935 (0.738, 1.196) | 0.95 (0.791, 1.141) |

**APPENDIX 1B:** Adjusted consumption frequency ratios for individual foods comparing food insecure at any point vs. Persistently food secure, by program participation

|  | **Estimated Frequency Ratio, Food Insecure at Any Point vs. Persistently Food Secure (95% CrI)** | | | |
| --- | --- | --- | --- | --- |
| Food item | No SNAP or Pantry | Pantry, no SNAP | SNAP, no Pantry | Both Pantry and SNAP |
| **Healthy items** |  |  |  |  |
| *Fruit* | **0.739 (0.606, 0.897)** | 1.134 (0.666, 1.943) | **0.728 (0.542, 0.977)** | 1.117 (0.670, 1.861) |
| *Vegetables* | **0.693 (0.571, 0.842)** | 1.238 (0.716, 2.096) | **0.656 (0.491, 0.875)** | 1.173 (0.712, 1.921) |
| *Whole Grains* | **0.781 (0.635, 0.966)** | 1.132 (0.638, 2.007) | 0.950 (0.691, 1.300) | 1.377 (0.807, 2.389) |
| *Nuts* | **0.792 (0.643, 0.979)** | 1.382 (0.796, 2.420) | 0.917 (0.674, 1.257) | 1.600 (0.944, 2.694) |
| *Legumes* | 0.942 (0.780, 1.147) | 1.689 (0.983, 2.866) | 1.046 (0.785, 1.394) | 1.874 (1.136, 3.082) |
| *Fish* | 1.007 (0.857, 1.183) | 0.857 (0.540, 1.348) | 0.921 (0.726, 1.170) | 0.784 (0.513, 1.195) |
| **Unhealthy items** |  |  |  |  |
| *Processed Meats* | 1.019 (0.849, 1.225) | 0.981 (0.594, 1.657) | 0.927 (0.704, 1.216) | 0.892 (0.543, 1.459) |
| *Beef, Pork, Lamb* | **0.828 (0.689, 0.998)** | 0.993 (0.594, 1.662) | **0.634 (0.482, 0.832)** | 0.761 (0.471, 1.235) |
| *Fast Food* | 0.876 (0.732, 1.046) | 0.770 (0.461, 1.290) | 0.859 (0.655, 1.125) | 0.755 (0.464, 1.229) |
| *SSBs* | 0.853 (0.679, 1.068) | 1.195 (0.650, 2.220) | **0.659 (0.463, 0.929)** | 0.924 (0.511, 1.641) |
| *Sweets* | **0.738 (0.603, 0.904)** | 0.934 (0.529, 1.645) | 0.782 (0.568, 1.067) | 0.989 (0.575, 1.686) |
| *Refined Grains* | 0.865 (0.71, 1.062) | 1.105 (0.638, 1.913) | **0.724 (0.536, 0.980)** | 0.925 (0.544, 1.547) |
| *Full-Fat Dairy* | **0.769 (0.623, 0.940)** | 0.751 (0.436, 1.303) | 0.785 (0.577, 1.061) | 0.766 (0.457, 1.295) |
| *Alcohol* | 0.913 (0.768, 1.091) | 1.025 (0.638, 1.670) | 0.928 (0.706, 1.207) | 1.041 (0.660, 1.637) |

**APPENDIX 2:** Adjusted consumption frequency ratios for individual foods, by food pantry, SNAP, and food insecurity status


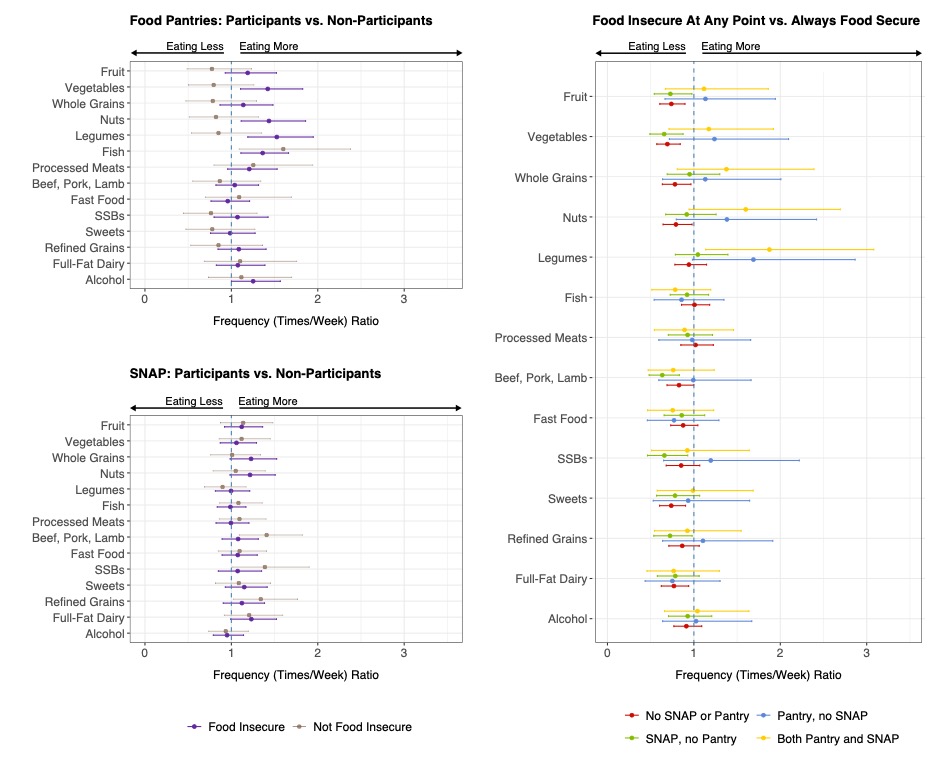


**APPENDIX 3:** Distribution, by weight, of foods shipped to partner food pantries by The Greater Boston Food Bank from January 1, 2020, through December 31, 2020^1^

| **Product** | **Percent of total weight shipped* (%)** |
| --- | --- |
| Fresh produce | 30% |
| Grains (rice, pasta, bread/bakery, cereal, etc.) | 10% |
| Mixed and assorted food items | 9% |
| Poultry | 8% |
| Dairy products | 7% |
| Non-meat protein | 7% |
| Complete meal/entree and soups | 6% |
| Juice 100% | 5% |
| Meat - not seafood or poultry | 5% |
| Fruit - canned/frozen | 4% |
| Vegetables - canned/frozen | 3% |
| Condiments/Sauces/Spices/Dressing | 3% |
| Seafood | 2% |
| Beverages - not water or soda | <1% |
| Snack Food/Cookies/Dessert | <1% |
| Soda | <1% |
| Non-dairy dairy substitutes | <1% |
| *Total weight shipped was 95 million pounds | |

^1^ Data in this table was provided via direct communication with the Greater Boston Food Bank.
